# Supplementary material for: Resolving the full spectrum of human genome variation using Linked-Reads
Source: Genome Res. 2019 Apr;29(4):635–45. doi: 10.1101/gr.234443.118 (PMC6442396; doi:10.1101/gr.234443.118)
Supplement: Supplemental Material [file supp_gr.234443.118_Supplemental_longranger-2.2.zip › longranger-2.2.tar.bz2!/lr2.2-public/lib/stan/cmdstan/stan/doc/api/html/statement__grammar__def_8hpp.html]

Stan: src/stan/gm/grammars/statement\_grammar\_def.hpp File Reference


|  |  |
| --- | --- |
|  | Stan  2.5.0  probability, sampling & optimization |


- Main Page
- Related Pages
- Namespaces
- Classes
- Files

- File List
- File Members

All Classes Namespaces Files Functions Variables Typedefs Enumerations Enumerator Friends Macros Pages

- src
- stan
- gm
- grammars

Classes |
Namespaces |
Functions |
Variables

statement\_grammar\_def.hpp File Reference

`#include <cstddef>`  
`#include <iomanip>`  
`#include <iostream>`  
`#include <istream>`  
`#include <map>`  
`#include <set>`  
`#include <sstream>`  
`#include <string>`  
`#include <utility>`  
`#include <vector>`  
`#include <stdexcept>`  
`#include <boost/spirit/include/qi.hpp>`  
`#include <boost/lexical_cast.hpp>`  
`#include <boost/fusion/include/adapt_struct.hpp>`  
`#include <boost/fusion/include/std_pair.hpp>`  
`#include <boost/config/warning_disable.hpp>`  
`#include <boost/spirit/include/qi_numeric.hpp>`  
`#include <boost/spirit/include/phoenix_core.hpp>`  
`#include <boost/spirit/include/phoenix_function.hpp>`  
`#include <boost/spirit/include/phoenix_fusion.hpp>`  
`#include <boost/spirit/include/phoenix_object.hpp>`  
`#include <boost/spirit/include/phoenix_operator.hpp>`  
`#include <boost/spirit/include/phoenix_stl.hpp>`  
`#include <boost/spirit/include/support_multi_pass.hpp>`  
`#include <boost/tuple/tuple.hpp>`  
`#include <boost/variant/apply_visitor.hpp>`  
`#include <boost/variant/recursive_variant.hpp>`  
`#include <boost/spirit/include/version.hpp>`  
`#include <boost/spirit/include/support_line_pos_iterator.hpp>`  
`#include <stan/gm/ast.hpp>`  
`#include <stan/gm/grammars/whitespace_grammar.hpp>`  
`#include <stan/gm/grammars/expression_grammar.hpp>`  
`#include <stan/gm/grammars/var_decls_grammar.hpp>`  
`#include <stan/gm/grammars/statement_grammar.hpp>`  
`#include <stan/gm/grammars/common_adaptors_def.hpp>`

Go to the source code of this file.

|  |  |
| --- | --- |
| Classes | |
| struct | stan::gm::validate\_return\_allowed |
|  | |
| struct | stan::gm::validate\_return\_allowed::result< T1, T2, T3 > |
|  | |
| struct | stan::gm::validate\_void\_return\_allowed |
|  | |
| struct | stan::gm::validate\_void\_return\_allowed::result< T1, T2, T3 > |
|  | |
| struct | stan::gm::validate\_assignment |
|  | |
| struct | stan::gm::validate\_assignment::result< T1, T2, T3, T4 > |
|  | |
| struct | stan::gm::validate\_sample |
|  | |
| struct | stan::gm::validate\_sample::result< T1, T2, T3 > |
|  | |
| struct | stan::gm::expression\_as\_statement |
|  | |
| struct | stan::gm::expression\_as\_statement::result< T1, T2, T3 > |
|  | |
| struct | stan::gm::unscope\_locals |
|  | |
| struct | stan::gm::unscope\_locals::result< T1, T2 > |
|  | |
| struct | stan::gm::add\_while\_condition |
|  | |
| struct | stan::gm::add\_while\_condition::result< T1, T2, T3 > |
|  | |
| struct | stan::gm::add\_while\_body |
|  | |
| struct | stan::gm::add\_while\_body::result< T1, T2 > |
|  | |
| struct | stan::gm::add\_loop\_identifier |
|  | |
| struct | stan::gm::add\_loop\_identifier::result< T1, T2, T3, T4 > |
|  | |
| struct | stan::gm::remove\_loop\_identifier |
|  | |
| struct | stan::gm::remove\_loop\_identifier::result< T1, T2 > |
|  | |
| struct | stan::gm::validate\_int\_expr2 |
|  | |
| struct | stan::gm::validate\_int\_expr2::result< T1, T2, T3 > |
|  | |
| struct | stan::gm::validate\_allow\_sample |
|  | |
| struct | stan::gm::validate\_allow\_sample::result< T1, T2, T3 > |
|  | |
| struct | stan::gm::validate\_non\_void\_expression |
|  | |
| struct | stan::gm::validate\_non\_void\_expression::result< T1, T2, T3 > |
|  | |

|  |  |
| --- | --- |
| Namespaces | |
|  | stan |
|  | Probability, optimization and sampling library. |
|  | |
|  | stan::gm |
|  | Directed graphical model compiler. |
|  | |

|  |  |
| --- | --- |
| Functions | |
|  | BOOST\_FUSION\_ADAPT\_STRUCT (stan::gm::assignment,(stan::gm::variable\_dims, var\_dims\_)(stan::gm::expression, expr\_)) |
|  | |
|  | BOOST\_FUSION\_ADAPT\_STRUCT (stan::gm::variable\_dims,(std::string, name\_)(std::vector< stan::gm::expression >, dims\_)) |
|  | |
|  | BOOST\_FUSION\_ADAPT\_STRUCT (stan::gm::distribution,(std::string, family\_)(std::vector< stan::gm::expression >, args\_)) |
|  | |
|  | BOOST\_FUSION\_ADAPT\_STRUCT (stan::gm::for\_statement,(std::string, variable\_)(stan::gm::range, range\_)(stan::gm::statement, statement\_)) |
|  | |
|  | BOOST\_FUSION\_ADAPT\_STRUCT (stan::gm::return\_statement,(stan::gm::expression, return\_value\_)) |
|  | |
|  | BOOST\_FUSION\_ADAPT\_STRUCT (stan::gm::print\_statement,(std::vector< stan::gm::printable >, printables\_)) |
|  | |
|  | BOOST\_FUSION\_ADAPT\_STRUCT (stan::gm::reject\_statement,(std::vector< stan::gm::printable >, printables\_)) |
|  | |
|  | BOOST\_FUSION\_ADAPT\_STRUCT (stan::gm::increment\_log\_prob\_statement,(stan::gm::expression, log\_prob\_)) |
|  | |
|  | BOOST\_FUSION\_ADAPT\_STRUCT (stan::gm::sample,(stan::gm::expression, expr\_)(stan::gm::distribution, dist\_)(stan::gm::range, truncation\_)) |
|  | |
|  | BOOST\_FUSION\_ADAPT\_STRUCT (stan::gm::statements,(std::vector< stan::gm::var\_decl >, local\_decl\_)(std::vector< stan::gm::statement >, statements\_)) |
|  | |

|  |  |
| --- | --- |
| Variables | |
| boost::phoenix::function  < validate\_return\_allowed > | stan::gm::validate\_return\_allowed\_f |
|  | |
| boost::phoenix::function  < validate\_void\_return\_allowed > | stan::gm::validate\_void\_return\_allowed\_f |
|  | |
| boost::phoenix::function  < validate\_assignment > | stan::gm::validate\_assignment\_f |
|  | |
| boost::phoenix::function  < validate\_sample > | stan::gm::validate\_sample\_f |
|  | |
| boost::phoenix::function  < expression\_as\_statement > | stan::gm::expression\_as\_statement\_f |
|  | |
| boost::phoenix::function  < unscope\_locals > | stan::gm::unscope\_locals\_f |
|  | |
| boost::phoenix::function  < add\_while\_condition > | stan::gm::add\_while\_condition\_f |
|  | |
| boost::phoenix::function  < add\_while\_body > | stan::gm::add\_while\_body\_f |
|  | |
| boost::phoenix::function  < add\_loop\_identifier > | stan::gm::add\_loop\_identifier\_f |
|  | |
| boost::phoenix::function  < remove\_loop\_identifier > | stan::gm::remove\_loop\_identifier\_f |
|  | |
| boost::phoenix::function  < validate\_int\_expr2 > | stan::gm::validate\_int\_expr2\_f |
|  | |
| boost::phoenix::function  < validate\_allow\_sample > | stan::gm::validate\_allow\_sample\_f |
|  | |
| boost::phoenix::function  < validate\_non\_void\_expression > | stan::gm::validate\_non\_void\_expression\_f |
|  | |

## Function Documentation

|  |  |  |  |
| --- | --- | --- | --- |
| BOOST\_FUSION\_ADAPT\_STRUCT | ( | stan::gm::assignment | , |
|  |  | (stan::gm::variable\_dims, var\_dims\_)(stan::gm::expression, expr\_) |  |
|  | ) |  |  |

|  |  |  |  |
| --- | --- | --- | --- |
| BOOST\_FUSION\_ADAPT\_STRUCT | ( | stan::gm::variable\_dims | , |
|  |  | (std::string, name\_)(std::vector< stan::gm::expression >, dims\_) |  |
|  | ) |  |  |

|  |  |  |  |
| --- | --- | --- | --- |
| BOOST\_FUSION\_ADAPT\_STRUCT | ( | stan::gm::distribution | , |
|  |  | (std::string, family\_)(std::vector< stan::gm::expression >, args\_) |  |
|  | ) |  |  |

|  |  |  |  |
| --- | --- | --- | --- |
| BOOST\_FUSION\_ADAPT\_STRUCT | ( | stan::gm::for\_statement | , |
|  |  | (std::string, variable\_)(stan::gm::range, range\_)(stan::gm::statement, statement\_) |  |
|  | ) |  |  |

|  |  |  |  |
| --- | --- | --- | --- |
| BOOST\_FUSION\_ADAPT\_STRUCT | ( | stan::gm::return\_statement | , |
|  |  | (stan::gm::expression, return\_value\_) |  |
|  | ) |  |  |

|  |  |  |  |
| --- | --- | --- | --- |
| BOOST\_FUSION\_ADAPT\_STRUCT | ( | stan::gm::print\_statement | , |
|  |  | (std::vector< stan::gm::printable >, printables\_) |  |
|  | ) |  |  |

|  |  |  |  |
| --- | --- | --- | --- |
| BOOST\_FUSION\_ADAPT\_STRUCT | ( | stan::gm::reject\_statement | , |
|  |  | (std::vector< stan::gm::printable >, printables\_) |  |
|  | ) |  |  |

|  |  |  |  |
| --- | --- | --- | --- |
| BOOST\_FUSION\_ADAPT\_STRUCT | ( | stan::gm::increment\_log\_prob\_statement | , |
|  |  | (stan::gm::expression, log\_prob\_) |  |
|  | ) |  |  |

|  |  |  |  |
| --- | --- | --- | --- |
| BOOST\_FUSION\_ADAPT\_STRUCT | ( | stan::gm::sample | , |
|  |  | (stan::gm::expression, expr\_)(stan::gm::distribution, dist\_)(stan::gm::range, truncation\_) |  |
|  | ) |  |  |

|  |  |  |  |
| --- | --- | --- | --- |
| BOOST\_FUSION\_ADAPT\_STRUCT | ( | stan::gm::statements | , |
|  |  | (std::vector< stan::gm::var\_decl >, local\_decl\_)(std::vector< stan::gm::statement >, statements\_) |  |
|  | ) |  |  |


---

[ Stan Home Page ]

*© 2011–2014,
Stan Development Team.*
